# Coronary plaque healing: a safety net or a hazard indicator?

**DOI:** 10.1007/s11239-025-03152-9

**Published:** 2025-07-18

**Authors:** Kyriakos Dimitriadis, Eleni Adamopoulou, Nikolaos Pyrpyris, Eirini Dri, Sofia Vaina, Eirini Beneki, Panagiotis Tsioufis, Alexandros Kasiakogias, Alexios Antonopoulos, Konstantinos Aznaouridis, Konstantina Aggeli, Konstantinos Tsioufis

**Affiliations:** 1 First Department of Cardiology, School of Medicine, National and Kapodistrian University of Athens, Hippokration General Hospital, Vas Sofias 114, Athens, 115 27 Greece; 2 Department of Cardiology, Lausanne University Hospital and University of Lausanne, Lausanne, 1005 Switzerland

**Keywords:** Acute coronary syndrome, Atherosclerotic plaque disruption, Atherosclerotic plaque healing, Coronary artery disease

## Abstract

Coronary atherosclerotic plaques can lead to acute coronary syndrome (ACS) occurrence through three main mechanisms: plaque rupture, plaque erosion and calcified nodule. Many destabilized plaques, however, do not cause cardiovascular events. Instead, thrombus formation is confined, lumen patency is preserved and the arterial wall is restored in a process termed as plaque healing. Early studies regarding coronary plaque healing used arterial specimens to determine its prevalence and histological characteristics. Advances in imaging modalities later enabled the implementation of in vivo studies, which have used optical coherence tomography (OCT) to identify the repaired plaques. They are visualized as lesions with a heterogeneous signal-rich layered or multilayered pattern and a distinct optical density from underlying plaque components. On one hand, plaque healing acts as a protective mechanism against myocardial infarction and unstable angina. On the other hand, the presence of layered plaques indicates previous plaque destabilization and therefore increased cardiovascular risk. Clinicians ought to bear these in mind in order to better apply patient risk stratification and adjust medical interventions. The aim of this review is to discuss the physiology of coronary plaque healing, determine its prevalence and clinical significance, as well as propose possible pathophysiological mechanisms behind impaired plaque healing along with therapeutic options.

## Graphical Abstract

**Figure:**
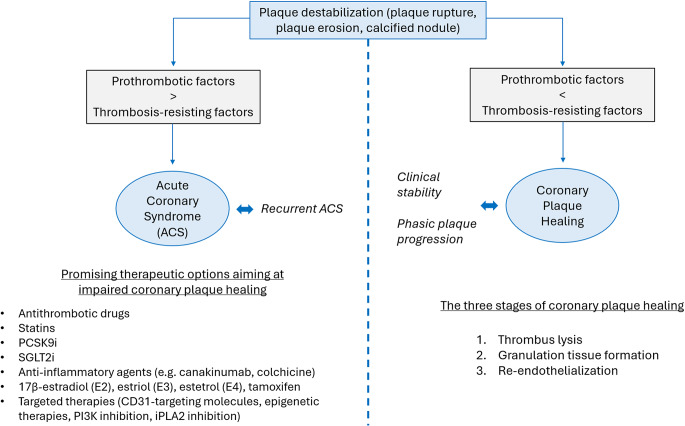
A significant amount of coronary atherosclerotic plaque destabilizations do not cause clinical events. The balance between prothrombotic and thrombosis-resisting factors will orientate either towards plaque healing or initiation of an ACS. When plaque healing occurs, it preserves lumen patency and prevents from ACS occurrence. It consists of three phases, namely thrombus lysis, granulation tissue formation and vessel re-endothelialization. Adequate plaque healing shifts CAD patients to a more stable phenotype, less “vulnerable” to acute events. A few drugs are promising options against impaired plaque healing and future studies should further investigate their potential in this context. Abbreviations: iPLA = calcium-independent phospholipase A2, PCSK9i = proprotein convertase subtilisin/kexin type 9 inhibitors, PI3K = phosphatidylinositol 3-kinase, SGLT2i = sodium-glucose co-transporter 2 inhibitors

## Introduction

Coronary artery disease (CAD) remains a leading cause of morbidity and mortality worldwide. It is now well known that atherosclerotic plaques can cause an acute coronary syndrome (ACS) by three main mechanisms: plaque rupture, plaque erosion and calcified nodules. However, not all plaque disruptions cause a clinical event. In fact, many of them remain quiescent as they heal and stabilize in a process termed “coronary plaque healing” [1, 2]. Plaque healing initiation mainly depends on the balance of pro-thrombotic and thrombosis-resisting factors. When thrombosis-resisting factors prevail, thrombus is retained, lumen patency is preserved and new tissue is formed, preventing ACS [3].

Early autopsy studies on patients having died from coronary events estimate the prevalence of healed plaques to be up to 61-73% [1, 2]. Advances in imaging technologies have enabled the in vivo identification of healed plaques. OCT is the gold-standard imaging modality for their visualization, as it has been validated against histopathology (81% sensitivity and 98% specificity for healed plaque identification) [4]. A few recent studies have utilized OCT to investigate the role of healed plaques, emphasizing on their prevalence, plaque characteristics and clinical importance.

Even though research efforts have mainly focused on the mechanisms of plaque instability, the notion that coronary plaque healing plays a determinant role in CAD has lately been gaining interest. Indeed, understanding the underlying causes of impaired plaque healing, as well as identifying potential therapeutic targets, is crucial for improving outcomes in this group of patients, especially in those with recurrent ACS. The aim of this review is to elucidate the main mechanisms of coronary plaque healing physiology, identify its clinical implications and role in CAD progression and ACS occurrence, as well as propose potential therapeutic strategies aimed at enhancing plaque healing patient capacity.

## Atherosclerotic plaque formation

Atherosclerosis has been found more likely to develop in specific sites of the arteries, the so-called “predilection sites”, that are characterized by low or oscillatory endothelial shear stress and are located near branch points or curvatures [5]. Evidence, however, regarding the initial trigger or triggers for atherogenesis is still scant [6]. From what is known and among other alterations, a qualitative change in the monolayer of endothelial cells (ECs) [7] as well as adaptive intimal thickening [8] occurs, constituting a transitional point for initial lesion development. The endothelium starts expressing adhesion molecules for leukocytes, while it becomes more permeable to low-density lipoprotein (LDL) particles, which then accumulate in the arterial wall [9]. LDL particles are then modified through oxidation and aggregation [10]. These modified LDLs cause ECs and vascular smooth muscle cells (VSMCs) to express adhesion molecules, chemo-attractants and growth factors that stimulate monocytes’ differentiation into macrophages and dendritic cells [5]. The macrophages then engulf LDLs via the scavenger receptors and become the so-called lipid-rich “foam cells”, named after their microscopic appearance [11]. In the beginning, foam cells accumulate in the proteoglycan layer of the intima, but then several layers form and ultimately create visible yellow-coloured xanthomas or fatty streaks. Some of the xanthomas accumulate acellular, lipid-rich material in the intima and turn into atherosclerotic lesions [5]. The scavenger receptors are not down-regulated by cellular cholesterol accumulation and therefore lead to constant LDL internalisation by the foam cells. This inevitably causes their apoptosis and the creation of a lipid-rich “necrotic core” within the atherosclerotic plaque [11]. Meanwhile, VSMCs undergo phenotypic modification from a contractile to a synthetic type [12] and produce a fibrous matrix composed of collagen, elastin and proteoglycans, which covers the plaque and is known as the “fibrous cap” [5].

## Atherosclerotic plaque disruption

There are three recognized acute coronary syndrome (ACS) mechanisms: plaque rupture, plaque erosion and calcified nodules. In vivo studies have shown that their incidence is 43.7–64.3%, 25.6–31.0% and 4.0-7.9% respectively, as identified by IVUS and OCT [13]. Plaque characteristics are the main determinants of the final mechanism [14].

Plaque rupture is characterized by a structural defect in the fibrous cap that leads to exposure of the lipid-rich necrotic core to the circulating blood. The highly thrombogenic substances of the necrotic core result in thrombus formation. Usually, the fibrous cap is thin and overlies a large necrotic core (thin-cap fibroatheroma, TCFA). Complex inflammatory mechanisms contribute to plaque rupture mainly by thinning of the fibrous cap [6, 14, 15].

In plaque erosion a discontinuity of the intimal endothelial lining can be found, exposing the underlying VSMCs, proteoglycans and glycosaminoglycans in the arterial lumen. The fibrous cap remains intact and the inflammatory mediators are less abundant in comparison with plaque rupture sites [14, 16, 17]. Plaque erosion has more favorable clinical, laboratory and angiographic characteristics against plaque rupture [16], leading to a better prognosis with fewer major adverse cardiovascular events (MACE) [18].

Calcified nodules are characterized as nodular calcifications extending into the lumen, with a disrupted fibrous cap [15, 19]. They are typically found in older patients and in cases involving highly calcified arteries [15]. A multicenter, prospective observational study has identified calcified nodules as the leading cause of acute coronary syndrome (ACS) with the highest 1-year incidence of MACE (32.1% in calcified nodules, compared to 12.4% in plaque rupture and 6.2% in plaque erosion, log-rank *p* < 0.0001) [20]. Torii et al. [21] supports the theory that the disruption of the fibrous cap in calcified nodules is triggered by fragmentation of the necrotic core calcifications, which is induced by the mechanical stress from surrounding stable, hard fibrocalcific plaques.

## Coronary plaque healing

Plaque disruption does not necessarily result in ACS. Interestingly, the majority of plaques rupture without associated symptoms or events [22]. In these cases, the thrombus formation is prevented or reversed at an early stage, the disrupted plaque is repaired and the lumen patency is preserved in a process known as atherosclerotic plaque healing. Healed plaques are therefore morphologically characterized by a single or multi-layered pattern that results from one or more silent episodes of plaque rupture, erosion or calcified nodules with a superimposed non-occlusive new fibrous layer formation [3, 23, 24].

### Physiology of coronary plaque healing

**Fig. 1:**
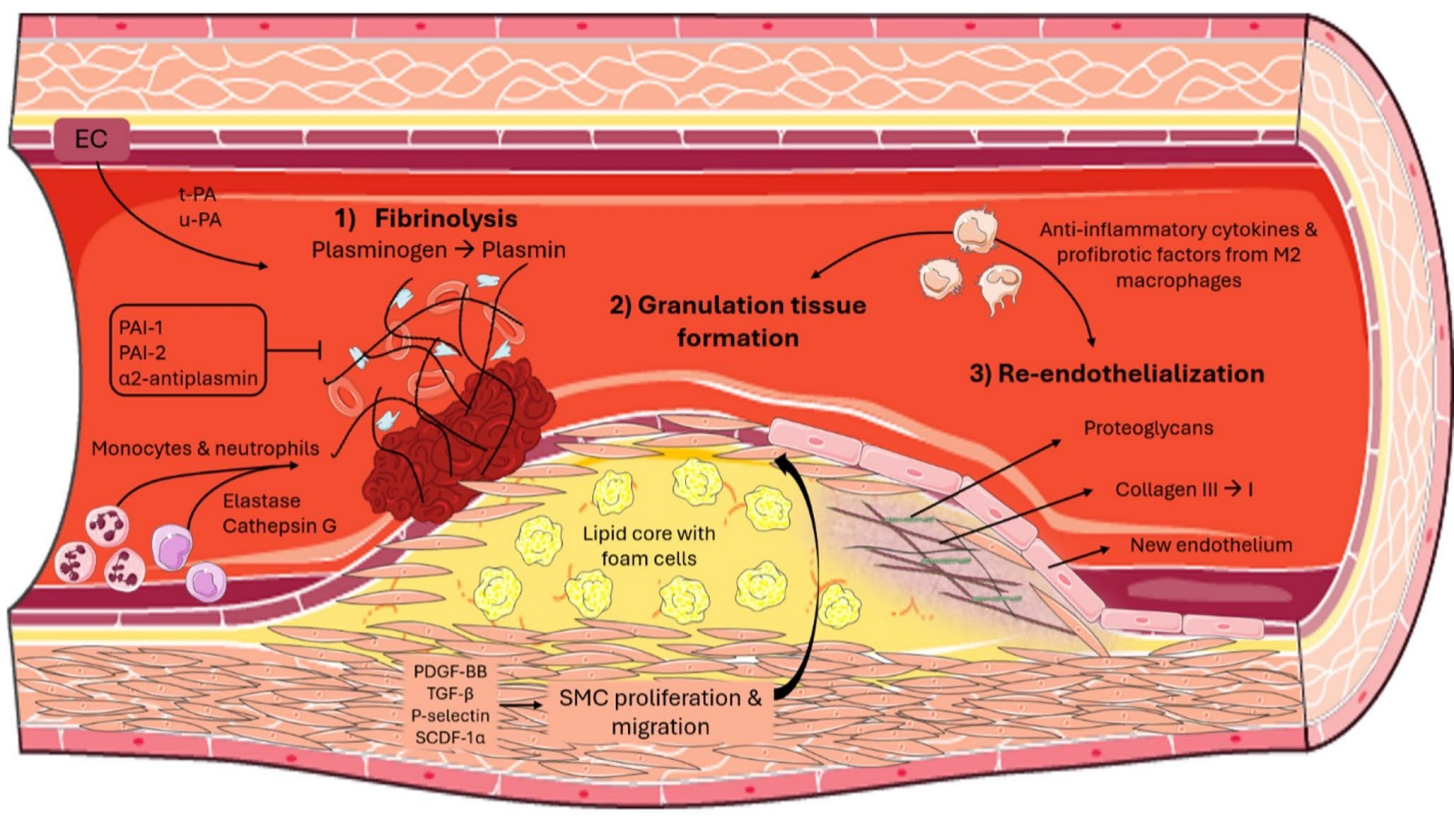
Phases of coronary plaque healing. Coronary plaque healing consists of three phases, namely thrombus lysis, granulation tissue formation and vessel re-reendothelialization, in addition to parallel anti-inflammatory pathways. Abbreviations: EC = endothelial cell, PAI-1 = plasminogen activator inhibitor 1, PAI-2 = plasminogen activator inhibitor 2, PDGF-BB = platelet-derived growth factor BB, SCDF-1α = stromal cell-derived factor 1α, SMC = smooth muscle cells, TGF-β = transforming growth factor β, t-PA = tissue plasminogen activator, u-PA = urokinase plasminogen activator

**Fig. 2:**
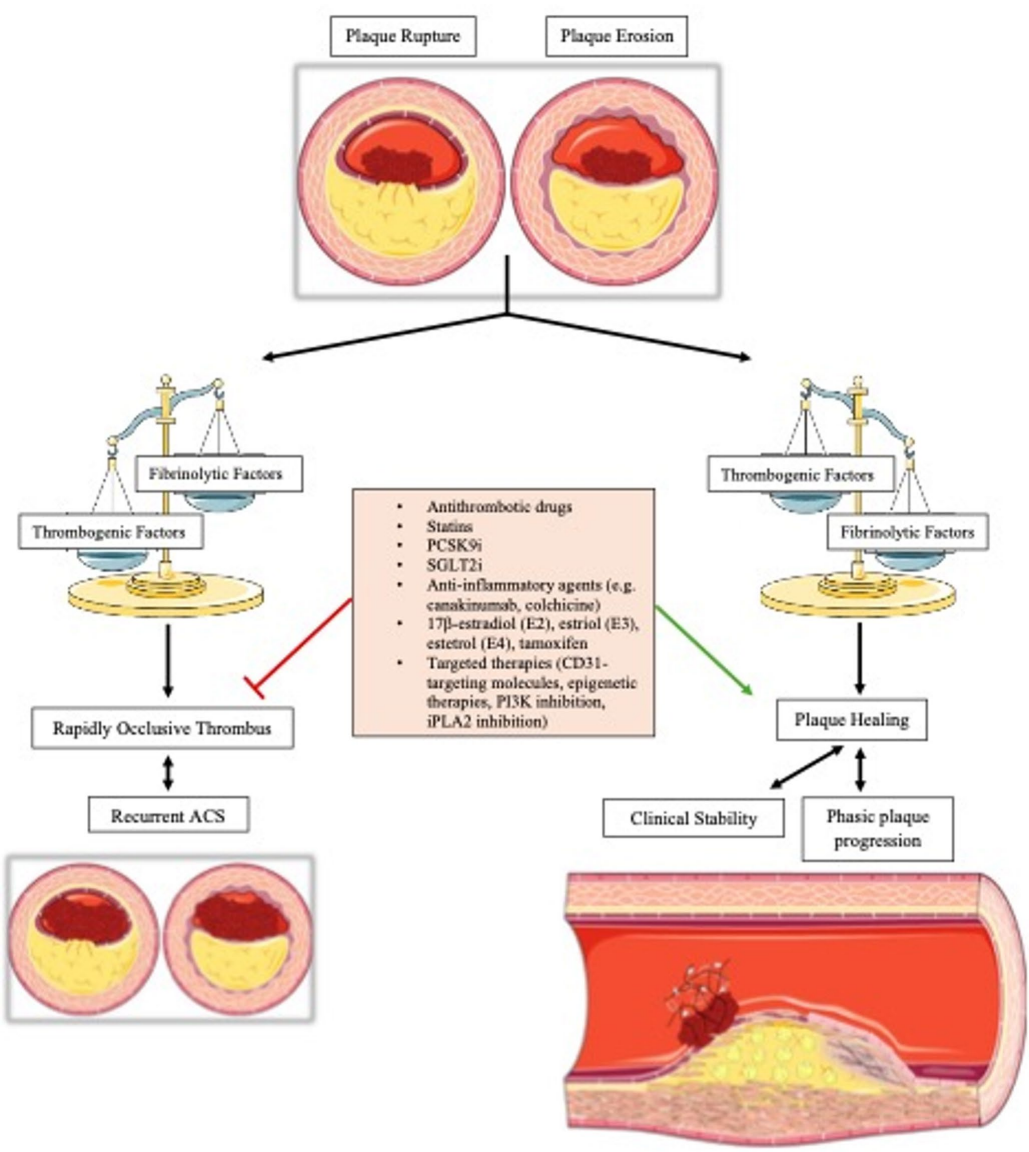
Natural history of plaque disruption. When an atherosclerotic plaque ruptures or erodes, the balance between prothrombotic and thrombosis-resisting factors will orientate either towards thrombus lysis, namely the first step of plaque healing, or the initiation of an ACS. Healed coronary plaques are rarely present in patients with recurrent ACS, as opposed to patients with long-standing stable angina pectoris [50]. In other words, the plaque healing process promotes clinical stability. At the same time, it is also a mechanism of phasic plaque progression, gradually reducing the luminal area. Promising therapeutic options (mentioned in the box at the center of the figure) may contribute to healing and clinical stability direction. Abbreviations: iPLA = calcium-independent phospholipase A2, PCSK9i = proprotein convertase subtilisin/kexin type 9 inhibitors, PI3K = phosphatidylinositol 3-kinase, SGLT2i = sodium-glucose co-transporter 2 inhibitors

According to Vergallo and Crea [3], atherosclerotic plaque healing consists of three phases, namely thrombus lysis, granulation tissue formation and vessel re-endothelialization, in addition to parallel anti-inflammatory pathways **(**Fig. 1**).** The exposure of thrombogenic plaque components after plaque rupture or erosion leads to platelet activation and thrombus formation [25], but at the same time endogenous fibrinolysis starts in order to preserve vessel patency [26]. Like dermal wound healing, the first step of hemostasis activation in order to form a platelet-fibrin scaffold is necessary up to a point, but in the end the balance between prothrombotic and thrombosis-resisting factors will orient either towards thrombus lysis, which is the first step of plaque healing, or the initiation of an ACS **(**Fig. 2**)**. Aside from normal thrombogenicity, an intact fibrinolytic system is also a prerequisite for healing initiation. Fibrinolysis is mediated mainly by tissue plasminogen activator (tPA), urokinase plasminogen activator (uPA), serine protease inhibitors, including α2-antiplasmin and plasminogen activator inhibitors 1 and 2 [27], elastase, cathepsin G and laminar blood flow [3].

If the thrombus lysis has occurred successfully, the second phase of plaque healing begins, namely granulation tissue formation. Smooth muscle cells (SMCs) from the media layer of the vessel and bone marrow-derived SMC progenitors from circulating blood proliferate and migrate to the intima layer [3]. This cell migration is mediated by growth factors like platelet-derived growth factor BB (PDGF-BB) and transforming growth factor β (TGF-β) [28], in addition to increased expression of P-selectin and stromal cell-derived factor 1α (SCDF-1α) [29]. The SMCs are then stimulated to produce an extracellular matrix rich in proteoglycans and type III collagen. Finally, in the third phase, collagen III is replaced by collagen I and re-endothelialization occurs [3].

### Necropsy studies regarding healed coronary plaques

The frequency of asymptomatic coronary atherosclerotic plaque disruption has been estimated to be 13% [30], 15% [31] and 31% [32]. Burke et al. [1] examined 142 male hearts from patients who experienced sudden coronary death and found that healed ruptures were present in 61% of cases. Healed plaques were associated with healed myocardial infarction (MI), elevated heart weight, diabetes and dyslipidemia, while increased number of healed rupture sites was observed in specimens with increased percent area luminal narrowing, supporting the hypothesis of repeated plaque rupture and healing as a mechanism of lumen stenosis advancement. This phasic, rather than linear, progression of CAD resulting from plaque disruption and healing is also supported by the study of Mann et al. [2], who estimated the prevalence of plaque healing at 16.2% in plaques with diameter stenosis of 0–20%, 18.6% in diameter stenosis of 21–50% and 73.2% in diameter stenosis > 50%. Moreover, in the histological part of the study by Okamoto et al. [33] it was shown that, among OCT-characterized heterogenous plaques of stable CAD patients, the layered ones had a significantly higher prevalence of intramural thrombus and macrophage infiltration as opposed to non-layered ones. This observation is in alignment with the following in vivo studies that also support the concept that healed plaques have vulnerable characteristics.

### In vivo studies regarding healed coronary plaques

**Fig. 3:**
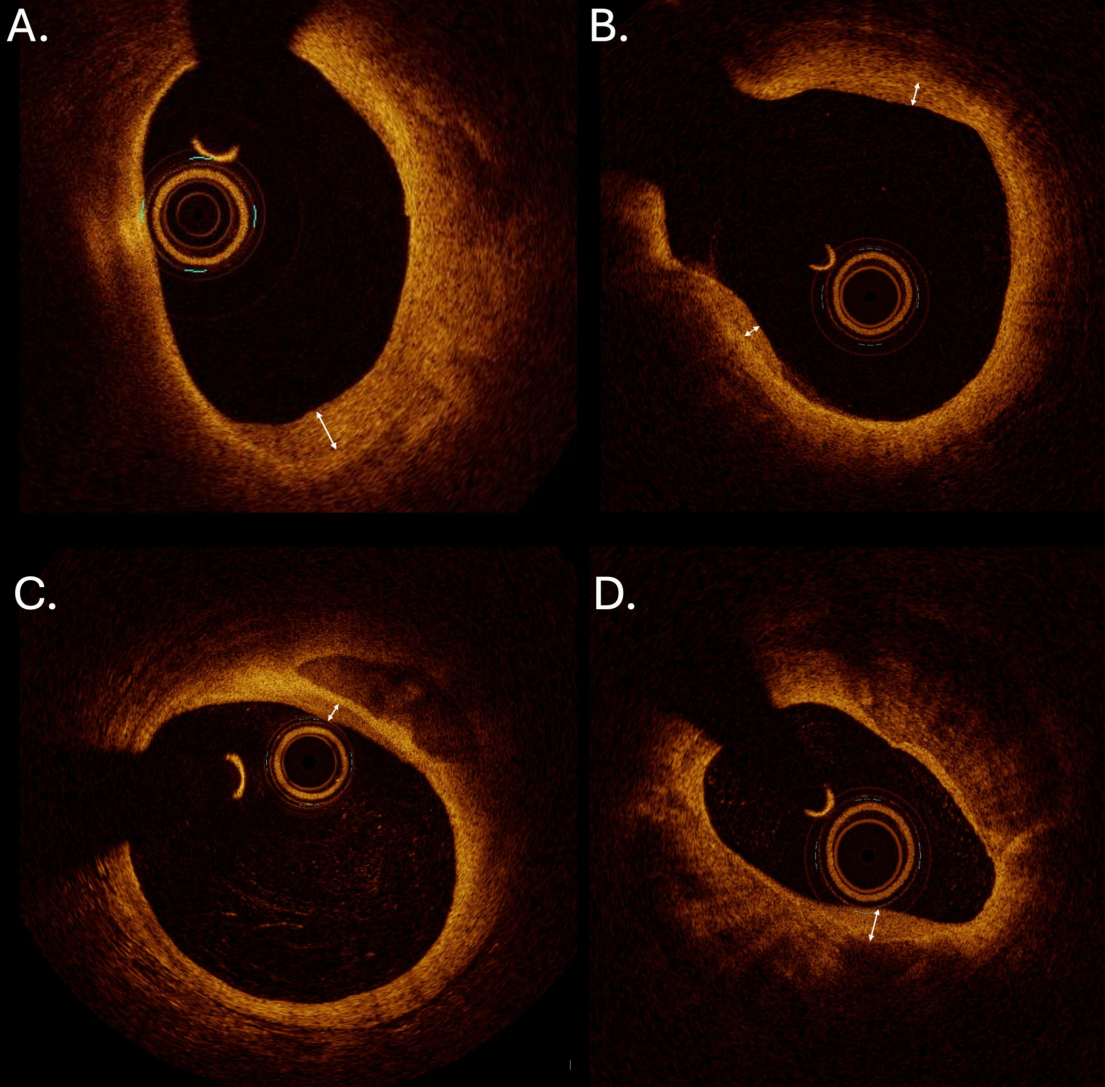
Representative OCT images showing coronary artery plaques with a layered pattern. Healed plaques in OCT can be seen as lesions with a heterogeneous signal-rich multilayer pattern and a distinct optical density from underlying plaque components, having a characteristic “onion-like” appearance (white arrows in panels A-D)

More recent studies have deployed the advancements of new imaging modalities to evaluate the prevalence and role of plaque healing in vivo. Optical coherence tomography (OCT) is the gold-standard method to visualize healed plaques due to its high resolution. Healed plaques in OCT can be seen as lesions with a heterogeneous signal-rich multilayer pattern and a distinct optical density from underlying plaque components **(**Fig. 3**)** [23, 24, 34, 35]. For this reason, the terms “healed” and “layered” plaques are used interchangeably in many studies. Shimokado et al. [4] estimated the sensitivity, specificity, positive and negative predictive value of OCT-defined healed coronary plaques at 81%, 98%, 93% and 93%, respectively. Recently, identification of layered plaques in OCT coronary images by deep learning has exerted promising results, constituting a possible future valuable tool [36].

#### Prevalence and incidence

In patient-level analysis, the prevalence of healed plaques in all three main coronary arteries has been estimated at 77% in patients with stable CAD [4] and 74.5% in acute MI patients (10.8% with only culprit layered plaques, 34.2% with only non-culprit layered plaques and 29.5% with both culprit and non-culprit layered plaques) [37]. Kurihara et al. [38] investigated the prevalence of healed plaques in ACS and stable CAD patients only on the culprit vessel and estimated it at 36.2%, with stable CAD patients exhibiting a significantly higher prevalence than ACS patients (57.8% vs. 25.1%, *p* < 0.001). One study has calculated the incidence of plaque healing at the culprit plaque erosion site of ACS patients at 55.5% after one month and at 69.2% after one year [39].

In plaque-level analysis, the prevalence of healed plaques at the culprit lesion of ACS patients has been reported as 29% [23] and 40.3% [37], while at non-culprit lesions as 35.5% [24] and 34.3% [37]. In culprit lesions of stable CAD patients the corresponding proportion has been estimated at 36.6% [33]. No significant difference has been observed in the prevalence of layered plaques between culprit ruptures and erosions in ACS patients [37]. Intriguingly, Nishi et al. [34] demonstrated that the prevalence of layered pattern is significantly higher in plaques located at vessels that display spasm spontaneously or after acetylcholine provocation test compared to vessels that do not undergo spasm (93% vs. 38%, *p* < 0.001). Last but not least, one study has estimated that 14.8% of non-culprit plaques develop a new layered pattern within one year after ACS [40].

#### Baseline patient characteristics

In the study of Fracassi et al. [23], ACS patients with healed culprit plaques had increased prevalence of hyperlipidemia, diabetes and history of MI. Dai et al. [37] demonstrated that pre-infarction angina, STEMI, higher LDL and absence of antiplatelet therapy are independent predictors of layered culprit plaques in acute MI patients, while Okamoto et al. [33] showed that younger age and smoking are independently associated with layered plaques in stable CAD patients. Another study has identified high triglyceride levels as an independent factor for the presence of layered non-culprit plaques in stable CAD patients [41]. Regarding sex, Seegers et al. report no difference in layered culprit plaque prevalence between men and women with stable CAD [42].

#### Characteristics of healed plaques

Studies researching on the characteristics of healed plaques are summarized in Table 1. In ACS patients, both culprit and non-culprit layered plaques tend to cluster in the proximal segment of the left anterior descending artery (LAD) and the left circumflex artery (LCx), whilst being more evenly distributed in the right coronary artery (RCA). Their frequency increases with increasing lumen area stenosis [23, 37]. Healed culprit plaques are more frequently TCFAs or lipid plaques and more likely to present with plaque rupture and complex B2/C type according to AHA/ACC classification [23]. Furthermore, they are characterized by larger lipid index [43], longer lipid core length, display a higher prevalence of microchannels and calcification [37], as well as cholesterol crystals and thrombi [44]. Yuki et al. has also found greater IVUS-defined total atheroma volume, percent atheroma volume and plaque burden [43]. Healed plaque subgroup analysis has revealed that multilayered plaques, as opposed to single-layered, have a higher prevalence of complex B2/C type lesions, display increased area stenosis [23] and increased percent atheroma volume [43].

**Table 1:** Studies investigating the characteristics of healed plaques with OCT or IVUS

Study	Number of patients	Observed characteristics of layered compared to non-layered plaques
**ACS, Culprit Plaques**		
Fracassi et al. 2019 [23]	376 patients, 376 culprit plaques, 108 layered culprit plaques	• ↑ Plaque rupture (%) (64.8 vs. 53.0, *p* = 0.039) • ↑ Lipid plaque (%) (83.3 vs. 70.9, *p* = 0.013) • ↑ TCFA (%) (56.5 vs. 42.5, *p* = 0.016) • ↑ Macrophage accumulation (%) (81.1 vs. 63.4, *p* = 0.001) • ↑ Area stenosis (%) (79.2 ± 9.5 vs. 74.3 ± 14.3, *p* = 0.001)
Dai et al. 2020 [37]	325 patients, 325 culprit plaques, 131 patients with layered culprit plaques	• ↑ Lipid core length (mm) [16.9 (15.2–18.6) vs. 14.5 (13.3–15.7), *p* = 0.023] • ↑ Macrophage accumulation (%) (96.9 vs. 78.4, *p* < 0.001) • ↑ Microchannels (%) (67.2 vs. 51.0, *p* = 0.004) • ↑ Calcification (%) (52.7 vs. 34.5, *p* = 0.001) • ↑ Thrombus (%) (90.1 vs. 77.3, *p* = 0.004) • ↓ MLA (mm^2^) [1.8 (1.6-2.0) vs. 2.2 (2.0-2.4), *p* = 0.008]
Yuki et al. 2023 [43]	150 patients, 52 patients with layered culprit plaques	• ↑ Lipid index (mm°) [1958.0 (420.9-2502.7) vs. 597.2 (169.1-1624.7), *p* = 0.014] • ↑ TAV (mm^3^) [183.3 (114.2–275.0) vs. 119.3 (68.9-185.5), *p* = 0.004] • ↑ PAV (%) [60.1 (54.7–60.1) vs. 53.7 (46.8–60.6), *p* = 0.001] • ↑ PB (%) [86.5 (81.7–85.7) vs. 82.6 (77.9–85.4), *p* = 0.001] • ↓ Spotty calcium (%) (40.4 vs. 59.2, *p* = 0.031)
Yao et al. 2022 [44]	233 patients, 89 patients with layered culprit plaques	• ↑ Lipid-rich plaque (%) (98.9 vs. 72.9, *p* < 0.001) • ↑ TCFA (%) (24.7 vs. 7.6, *p* < 0.001) • ↓ FCT (µm) (87.5 ± 33.5 vs. 109.4 ± 44.0, *p* < 0.001) • ↑ Lipid length (mm) (8.7 ± 3.4 vs. 6.3 ± 2.7, *p* < 0.001) • ↑ Max lipid arc (°) (264.1 ± 80.1 vs. 230.1 ± 69.1, *p* < 0 0.002) • ↑ Mean lipid arc (°) (204.9 ± 60.5 vs. 188.4 ± 48.2, *p* = 0.035) • ↑ Lipid index (mm°) (1854.3 ± 1060.0 vs. 1226.7 ± 710.3, *p* < 0.001) • ↑ Calcification (%) (60.7 vs. 41.0, *p* < 0.005) • ↑ Cholesterol crystals (%) (37.1 vs. 8.3, *p* < 0.001) • ↑ Macrophage accumulation (%) (74.2 vs. 32.6, *p* < 0.001) • ↑ Thrombus (%) (23.6 vs. 9.0, *p* = 0.004) • ↑ Plaque rupture (%) (64.8 vs. 53.0, *p* < 0.001) • ↓ MLA (mm^2^) (2.3 ± 1.3 vs. 3.1 ± 2.0, *p* < 0.001) • ↑ Area stenosis (%) (76.9 ± 11.3 vs. 69.5 ± 14.1, *p* < 0.001)
**ACS**,** Non-Culprit Plaques**		
Russo et al. 2019 [24]	349 patients, 165 layered non-culprit plaques, 300 non-layered non-culprit plaques	• ↓ Fibrous plaque (%) (6.7 vs. 14.0, *p* = 0.028) • ↑ Lipid plaque (%) (93.3 vs. 86.0, *p* = 0.028) • ↓ FCT (mm) (100.9 ± 42.6 vs. 114.2 ± 47.4, *p* = 0.008) • ↑ Lipid length (mm) (12.9 ± 6.1 vs. 10.6 ± 5.8, *p* < 0.001) • ↑ Lipid index (mm°) (2618.5 ± 1591.1 vs. 2057.7 ± 1401.8, *p* = 0.001) • ↑ Plaque length (mm) (16.7 ± 7.1 vs. 15.0 ± 7.2, *p* = 0.017) • ↑ TCFA (%) (29.7 vs. 13.7, *p* < 0.001) • ↑ Macrophages (%) (82.4 vs. 54.0, *p* < 0.001)
Dai et al. 2020 [37]	325 patients, 1136 non-culprit plaques, 390 patients with layered non-culprit plaques	• ↑ Lipid core length (mm) [11.2 (10.2–12.2) vs. 9.5 (8.9–10.1), *p* = 0.003] • ↑ Macrophage accumulation (%) (89.2 vs. 79.6, *p* < 0.001) • ↑ Microchannels (%) (65.6 vs. 51.7, *p* < 0.001) • ↑ Calcification (%) (47.2 vs. 37.9, *p* = 0.003) • ↓ MLA (mm^2^) [3.4 (3.2–3.7) vs. 4.3 (4.1–4.5), *p* < 0.001] • ↑ Area stenosis (%) [57.4 (55.7–59.1) vs. 48.4 (47.1–49.8), *p* < 0.001]
**Stable CAD**,** Culprit Plaques**		
Okamoto et al. 2019 [33]	205 patients, 75 patients with layered culprit plaques	• ↑ Microchannels (%) (57.3 vs. 15.4, *p* < 0.05) • ↓ MLA (mm^2^) (1.5 ± 0.9 vs. 1.9 ± 1.0, *p* < 0.05)
Matsuo et al. 2018 [45]	150 patients	• ↑ Area stenosis (*p* < 0.001): o Q1 (area stenosis ≥ 77.0%) ◊ 63% prevalence of healed plaques o Q2 (area stenosis 65.1–76.9%) ◊ 48% prevalence of healed plaques o Q3 (area stenosis 53.1–65.0%) ◊ 38% prevalence of healed plaques o Q4 (area stenosis ≤ 53.0%) ◊ 25% prevalence of healed plaques
Shimokado et al. 2018 [4]	60 patients, 46 patients with layered culprit plaques	• ↑ Macrophage accumulation (%) (70 vs. 21, *p* < 0.01) • ↑ Microchannels (%) (43 vs. 0, *p* < 0.01)
Russo et al. 2020 [46]	163 patients, 87 patients with layered culprit plaques	• ↓ Fibrous plaque (%) (16.1 vs. 35.5, *p* = 0.004) • ↑ Lipid plaque (%) (83.9 vs. 64.5, *p* = 0.004) • ↑ Lipid length (mm) (13.0 ± 8.0 vs. 9.1 ± 5.1, *p* = 0.001) • ↑ Lipid index (mm°) (2503.0 ± 1669.4 vs. 1648.2 ± 1105.8, *p* = 0.001) • ↑ Macrophage accumulation (%) (58.6 vs. 35.5, *p* = 0.003) • ↑ Calcification (%) (78.2 vs. 63.2, *p* = 0.035) • ↑ Large calcification (%) (62.1 vs. 40.8, *p* = 0.007) • ↑ Thrombus (%) (28.7 vs. 14.5, *p* = 0.029) • ↑ Plaque length (mm) (22.5 ± 10.6 vs. 16.3 ± 6.8, *p* < 0.001 • ↑ Area stenosis (%) (77.5 ± 11.4 vs. 73.1 ± 11.1, *p* = 0.015)
Kimura et al. 2021 [47]	33 patients, 16 patients with layered culprit plaques	• ↑TCFA (%) (31.6 vs. 0.0, *p* = 0.02) • ↑ Macrophage accumulation (%) (89.5 vs. 41.2, *p* = 0.004) • ↑Area stenosis (%) (79.6 ± 10.6 vs. 68.0 ± 21.6, *p* = 0.047)
Yao et al. 2022 [44]	157 patients, 97 patients with layered culprit plaques	• ↑ Fibrous plaque (%) (73.2 vs. 53.3, *p* = 0.018) • ↑ Lipid-rich plaque (%) (95.9 vs. 80, *p* = 0.003) • ↑ Lipid length (mm) (8.9 ± 3.2 vs. 6.9 ± 3.4, *p* = 0.001) • ↑ Lipid index (mm°) (1736.4 ± 869.4 vs. 1371.9 ± 968.6, *p* = 0.024) • ↑ Calcification (%) (71.1 vs. 48.3, *p* = 0.007) • ↑ Macrophage accumulation (%) (61.9 vs. 35.0, *p* = 0.002) • ↓ MLA (mm^2^) (2.2 ± 1.4 vs. 2.7 ± 1.7, *p* = 0.09) • ↑ Area stenosis (%) (77.0 ± 10.6 vs. 72.0 ± 12.4, *p* = 0.008)
Seegers et al. 2023 [42]	533 patients (418 men, 115 women), 292 patients with layered culprit plaques (230 men, 62 women)	In men: • ↑ Lipid plaque (%) (87 vs. 69, *p* < 0.001) • ↑ Lipid index (mm°) [1829 (1046–2851) vs. 1349 (878–2062), *p* < 0.001] • ↑ Mean lipid arc (°) (181 ± 46 vs. 169 ± 45, *p* = 0.022) • ↑ Macrophage accumulation (%) (69 vs. 56, *p* = 0.007) • ↑ Microchannels (%) (72 vs. 39, *p* < 0.001) • ↑ Cholesterol crystals (%) (49 vs. 30, *p* < 0.001) • ↑ Plaque rupture (%) (27 vs. 18, *p* = 0.031) • ↓ FCT (mm) (110 ± 64 vs. 126 ± 83, *p* = 0.050) • ↓ MLA (mm^2^) (1.5 ± 1 vs. 1.7 ± 1, *p* = 0.008) • ↑ Area stenosis (%) (79 ± 11 vs. 75 ± 11, *p* < 0.001) In women: no statistically significant differences were observed

***Abbreviations***: *ACS = acute coronary syndrome*,* CAD = coronary artery disease*,* FCT = fibrous cap thickness*,* MLA = minimal lumen area*,* PAV = percent atheroma volume*,* PB = plaque burden*,* TAV = total atheroma volume*,* TCFA = thin-cap fibroatheroma*

Non-culprit plaques with a layered pattern in ACS patients, compared to those without a layered pattern, are also characterized by more vulnerable features. More specifically, they are more likely to be lipid plaques and TCFAs, while also to have thinner fibrous cap, longer plaque and lipid length, greater lipid index [24] as well as higher prevalence of microchannels and calcification [37].

Similarly, in stable CAD patients, there is a graded increase in the prevalence of layered culprit plaques with an increase in the severity of area stenosis and a decrease in FFR [33, 45]. Patients with layered plaque phenotype at the culprit lesion have a higher prevalence of multivessel disease and more complex culprit lesions according to AHA/ACC classification. Furthermore, their layered culprit plaques have a higher prevalence of lipid plaque, calcifications, thrombus, microvessels as well as greater lipid burden, compared to non-layered ones [4, 46]. Kimura et al. additionally found increased prevalence of OCT-defined TCFAs and coronary angioscopy-defined red thrombus [47], while Yao et al. displayed increased fibrous plaques and longer lipid core length [44]. Seegers et al. aimed to investigate possible sex differences regarding healed culprit plaques in stable CAD patients and, even though they confirm that healed plaques exhibit more features of vascular inflammation and vulnerability in men, this observation was not confirmed in women [42]. Potential mechanisms for this difference include microvascular disease, endothelial dysfunction and plaque erosion [48], but more studies are needed in order to elucidate possible sex differences in the plaque healing process.

One study has examined non-culprit healed plaques in stable CAD patients. Non-culprit plaques in cases of layered culprit plaque, compared to cases of non-layered culprit plaque, have a higher prevalence of layered pattern and lipid plaque in addition to greater lipid burden and precent area stenosis. Irrespective of culprit lesion pattern, non-culprit plaques with a layered phenotype, compared to non-layered, have a higher prevalence of lipid plaque, cholesterol crystals, calcifications and spotty calcium, while they present with greater stenosis [46].

#### Inflammation

In ACS patients, increased macrophage accumulation has been observed more frequently in culprit plaques with a healed phenotype compared to non-healed [23, 37, 44], in non-culprit plaques in patients with a healed culprit plaque compared to non-healed culprit plaque, as well as in non-culprit healed plaques compared to non-culprit non-healed irrespective of culprit lesion morphology [24, 37] ACS patients with healed culprit plaques also have significantly higher levels of high-sensitivity C-reactive protein (hs-CRP) [23].

Similarly, in stable CAD patients, macrophages have been more frequently identified in culprit [4, 44, 46] and non-culprit [46] plaques with a healed phenotype compared to non-healed. Interestingly, in a retrospective observational study including stable CAD patients, layered plaques had significantly higher peri-coronary adipose tissue attenuation values than non-layered ones, indicating higher levels of vascular inflammation [49].

#### Clinical presentation

Vergallo et al. [50] demonstrated that healed coronary plaques, as depicted with OCT, are scarcely present in patients with a history of recurrent ACS, while their prevalence is significantly higher in patients with long-standing stable angina as well as in patients with a history of a single MI (3.3% vs. 29.7% vs. 28.9%, respectively, *p* = 0.01). Similarly, Wang et al. [35] showed that plaques with a multilayered pattern are more frequently observed in stable angina patients than ACS (75% vs. 51.4%, *p* = 0.001), indicating that adequate plaque healing shifts CAD patients to a more stable phenotype, less “vulnerable” to acute events. Likewise, in a retrospective single-center study, healed plaques were more frequently observed in patients with stable than unstable angina pectoris [44].

Meanwhile, Yamamoto et al. [51] has displayed that layered non-culprit plaques in stable CAD patients are characterized by a more rapid progression compared to non-layered plaques, as shown by greater increase in plaque cross-sectional area and plaque burden. Similarly, Yin et al. [52] identified the presence of healed non-culprit plaques in ACS patients as an independent predictor of subsequent lesion progression. These observations might indicate that, even though patients with healed plaques tend to have a more stable clinical presentation, they also have more accelerated stable CAD disease progression.

Whether ACS patients with layered plaques are more likely to present with non ST elevation MI (NSTEMI) or ST elevation MI (STEMI) remains uncertain, as the few available data provide contrasting and inconclusive results. Fracassi et al. [23] has demonstrated that ACS patients with a healed culprit plaque phenotype are more likely to present with NSTEMI than STEMI. However, Fang et al. [53] found similar prevalence of culprit layered plaque phenotype between STEMI and NSTEMI presentation, while the corresponding prevalence was higher in STEMI presentation when non-culprit plaques were examined. Median layer thickness and layer area were significantly larger in STEMI presentation both at culprit and non-culprit plaques.

#### Prognosis

Dai et al. [37] has demonstrated similar one-year MACE incidence between ACS patients with layered and non-layered plaques (both culprit and non-culprit). Likewise, in the study of Fracassi et al. [23], incidence of cardiac death, MI and ischemia-driven revascularization one year after ACS occurrence were similar between patients with and without healed culprit plaques. The rehospitalization rate, however, was higher in patients with layered culprit plaque. Moreover, a study including both ACS and stable CAD patients undergoing PCI showed that an OCT-detected untreated healed plaque in an non-culprit lesion is independently associated with increased incidence of non-culprit-related MACE both at patient and lesion level. The corresponding hazard ratios were 2.01 (*p* < 0.01) and 3.72 (*p* = 0.01) for patient and lesion level analysis, while MACE were defined as a composite of cardiac death, MI or ischemia-driven revascularization [54]. Another study that included both ACS and stable CAD patients found on multivariate Cox regression analysis that the presence of layered plaque on the culprit vessel is independently associated with increased risk of revascularization (HR 3.096, *p* = 0.026) [38]. Last but not least, when ACS patients were followed-up and grouped according to whether their non-culprit plaques developed a layered pattern, the 6-year incidence of non-culprit-related MACE was higher in the patients who developed the layered phenotype, mainly driven by increased rates of revascularization. The authors conclude that the creation of a new layered pattern might temporarily contribute to plaque stabilization but leads to vessel stenosis and worse outcomes [40]. The aforementioned studies regarding prognosis have many differences regarding the recruited CAD populations (stable, acute or both), the site of examined layered phenotype (culprit or non-culprit plaques) and the MACE definition. This heterogeneity might explain the diverse results, necessitating the conduction of larger studies for a safe conclusion to be drawn.

Taking into account all the aforementioned in vivo studies, it is evident that the presence of layered plaques may be associated with increased cardiovascular risk. Indeed, layered plaques have vulnerable characteristics and a few studies have demonstrated increased MACE incidence. It is important to note, however, that the presence of a layered plaque indicates that plaque destabilization had taken place prior to the layered pattern formation, meaning that the relevant atherosclerotic plaque probably already had high-risk features and the patient already was of increased cardiovascular risk. Thus, when patients with layered plaques are compared to their counterparts without this characteristic, it is reasonable to expect worse outcomes due to burdened history. The plaque healing process per se is a protective mechanism, without which patients would have had even worse outcomes.

### Impaired coronary plaque healing

#### Possible underlying mechanisms

The reason why some disrupted plaques heal and others do not remains unknown. Studies investigating the pathophysiology of impaired plaque healing are lacking. As stated before, after plaque disruption, the balance between prothrombotic and thrombosis-resisting factors will orient either towards thrombus lysis or the initiation of an ACS. Decreased plaque healing capacity could therefore result from a hypercoagulability or hypo-fibrinolysis state. This hypothesis is in line with the study of Lee et al. [55], who demonstrated that, among patients undergoing PCI, acute MI patients have higher platelet-fibrin clot strength, indicative of hypercoagulability, and lower fibrinolytic activity compared to non-AMI patients.

Recognized hypercoagulability mechanisms include increased platelet activation and aggregation, increased coagulation factors such as V, VII, VIII, XIII and von Willebrand, decreased anticoagulation factors including protein S and C, in addition to thrombogenic comorbidities like sickle cell disease, polycythemia, diabetes mellitus and increased anti-cardiolipin antibody– related diseases [56]. Blood thrombogenicity can be consistently high in some of the aforementioned cases, but a transient increase could also lead to acute MI [57, 58], as can occur due to smoking, cold stress, respiratory infection, mental stress and physical exertion [59].

Hypo-fibrinolysis is a recognized cause of thrombosis in many clinical entities, like type 2 diabetes [60] and antiphospholipid syndrome [61]. Interestingly, thin fibrin clots with a tight structure are dissolved slower than those with a loose structure made of thicker fibers [62], meaning that hypo-fibrinolysis related to defective fibrin architecture could also undermine the plaque healing process.

Future studies should focus more on identifying possible factors that undermine the coronary plaque healing process. For the time being, Russo et al. have provided the first evidence that air pollution might be one of them. More specifically, they demonstrated among patients with recurrent ACS that those with healed plaques have lower PM2.5 exposure levels compared to those without healed plaques [63].

#### Promising therapeutic options

Atherosclerotic plaque healing could be enhanced by targeting some of its mechanisms, mainly by reducing thrombotic potential, inflammation and oxidative stress, increasing fibrinolytic potential and collagen deposition, as well as altering VSMC phenotype. Potential therapeutic options are summarized in Table 2. Aspirin and P2Y_12_ receptor inhibitors display a protective role against ACS due to their inhibitory effects on platelet aggregation, however, they should not be administered on a routine basis for primary prevention due to increased bleeding risk [64, 65]. Whether patients with impaired plaque healing can benefit from antiplatelet drugs remains to be investigated. Statins, on the other hand, promote plaque healing via pleiotropic effects. They reduce platelet aggregation and thromboxane production [66], altering the levels of many thrombotic and fibrinolytic mediators, including coagulation factors V, VII, VIII, X, XI, vWF, protein C, tissue factor (TF), tissue factor pathway inhibitor (TFPI), fibrinogen, procoagulant phospholipids, anti-thrombin III, tPA, plasmin inhibitor, thrombin activatable fibrinolysis inhibitor (TAFI) and plasminogen activator inhibitors (PAIs) [67]. Statin use is associated with reduced D-dimer levels [68], as well as fibrin structure and function improvement, demonstrated by increased fibrin clot permeability (Ks) and decreased clot lysis time (CLT) [69]. As displayed in venous thromboembolism patients, statins reduce thrombin generation potential [70] and result in greater thrombus resolution or improvement [71]. A clinical trial on hypercholesterolemic patients has also shown that simvastatin reduces platelet aggregating responses to ADP, collagen, and arachidonic acid, as well as circulating levels of platelet markers [72]. Aside from the thrombosis– fibrinolysis system, novel therapeutic approaches could be developed to promote beneficial changes in VSMC phenotype and function [73]. Whether and how statins influence plaque healing by targeting VSMCs is still unknown, however evidence suggests their ability to inhibit proliferation [74, 75], migration [74–76] and apoptosis [77] of VSMCs. Data on the effect of statins on vascular collagen are conflicting, as some studies have demonstrated an increase [78] and others a decrease of its concentration [79]. On the contrary, data on statins’ effects on matrix metalloproteinases are more consistent, indicating a significant decrease in MMP1, MMP2, MMP3 and MMP9 [80–84]. Furthermore, proprotein convertase subtilisin/kexin type 9 (PCSK9) seems to also affect proliferation and migration of VSMCs [85] and PCSK9 inhibition leads to upregulation of pro-regenerative progenitor ECs [86]. Anti-inflammatory agents like canakinumab and colchicine, having already shown benefits in preventing recurrent ACS [87, 88], should be directly assessed regarding plaque healing capacity in future studies, as inflammation is a primary distinguisher between plaque instability and healing [3]. The same applies to sodium-glucose co-transporter 2 (SGLT2) inhibitors, which have already shown protective endothelial effects [89]. Interestingly, 17β-estradiol (E2) [90], estriol (E3), estetrol (E4) [91] and tamoxifen [92] are also able to accelerate endothelial healing.

**Table 2:** Potential therapeutic options for impaired plaque healing capacity

*Therapy*	*Plaque Healing Enhancement Mechanism*
Antiplatelet drugs (e.g. Aspirin, P2Y_12_ receptor inhibitors) [64, 65]	• Platelet aggregation inhibition
Lipid-lowering drugs (e.g. statins, PCSK9 inhibitors) [66–77, 80–86, 105]	• Platelet aggregation and thromboxane production reduction by altering the levels of thrombotic and fibrinolytic mediators • Fibrin structure and function improvement • Thrombus resolution • VSMCs proliferation, migration and apoptosis inhibition • Matrix metalloproteinases reduction (MMP1, MMP2, MMP3, MMP9) • Pro-regenerative progenitor ECs upregulation • Oxidative stress reduction • Inflammatory mediators reduction (e.g. macrophages)
Anti-inflammatory drugs (e.g. canakinumab, colchicine) [3, 87, 88]	• Adhesion molecules and leukocyte recruitment on ECs reduction • Thrombotic potential reduction • Fibrinolytic potential enhancement
SGLT2 inhibitors (e.g. empagliflozin, dapagliflozin) [89]	• Endothelial function improvement (increased NO production, mitochondrial homeostasis, endothelial cell viability, angiogenesis) • Oxidative stress and inflammation attenuation
17β-estradiol (E2) [90], estriol (E3), estetrol (E4) [91], tamoxifen [92]	• EC healing mediated by ERα and ERαMISS in ECs, as well as nuclear actions of ERα in SMCs
CD31-targeting molecules [93–95]	• Macrophage phenotype switching from the proinflammatory type to the reparative M2 type • Neutrophil activation limitation
Epigenetic therapies (e.g. DNA methylation, histone modifications, miRNAs, lnc-RNAs) [3, 96, 97]	• Macrophage phenotype switching from the proinflammatory type to the reparative M2 type
PI3K axis inhibitors [98, 99]	• EC migration across the injured area promotion by blocking the catalytic p110α, catalytic p110δ and regulatory p85α subunit isoforms of PI3K
iPLA2 inhibitors [100]	• EC migration across the injured area promotion through the lysoPC/TRPC6/Ca^2+^ pathway

***Abbreviations***: *ECs = endothelial cells*,* ERα = estrogen receptor α*,* ERαMISS = ERα membrane-initiated signalling*,* iPLA2 = calcium-independent phospholipase A2*,* lysoPC = lysophosphatidylcholine*,* MMPs = matrix metalloproteinases*,* NO = nitric oxide*,* PCSK9 = proprotein convertase subtilisin/kexin type 9*,* PI3K = phosphatidylinositol 3-kinase*,* SGLT2 = sodium-glucose co-transporter 2*,* SMCs = smooth muscle cells*,* TRPC6 = transient receptor potential cation channel subfamily C member 6*,* VSMCs = vascular smooth muscle cells*

Moving on to more targeted therapies, CD31-targeting molecules represent a novel option, as CD31 is a receptor found in leukocytes, platelets and endothelial cells, participating in the arterial healing process [93]. CD31 signaling promotes macrophage phenotype switching from the proinflammatory type to the reparative M2 type. This favors arterial healing, as shown in an animal model of acute aortic dissection and intramural hematoma [94], and limits neutrophil activation, as demonstrated in a model of acute mesenteric ischemia/reperfusion [95]. Macrophage polarization towards the M2 phenotype can also be achieved by epigenetic therapies, including DNA methylation, histone modifications, miRNAs and lnc-RNAs [3, 96, 97]. Last but not least, promoting EC migration across the injured area is another innovative target mechanism, as has been demonstrated by blocking the catalytic p110α, catalytic p110δ [98] and regulatory p85α [99] subunit isoforms of phosphatidylinositol 3-kinase (PI3K), as well as the calcium-independent phospholipase A2 (iPLA2) [100], through the lysoPC/TRPC6/Ca^2+^ pathway.

## Clinical implications and future directions

Based on the above, coronary plaque healing seems to play a crucial role in the event chain between plaque destabilization and ACS occurrence. Nevertheless, its significance in clinical practice remains to be fully acknowledged. The majority of research on ACS mechanisms has focused mainly on high-risk atherosclerotic plaque characteristics, including high plaque burden, low minimal lumen area and thin fibrous cap. However, the ability to predict ACS incidence relying only on atherosclerotic plaque characteristics remains weak, indicating the presence of additional contributing factors [101]. Impaired healing capacity after plaque disruption may constitute one of these additional factors, emphasizing the need to move away from the “vulnerable plaque” and orientate towards the more multifaceted concept of “vulnerable patient” [102]. Indeed, identifying a vulnerable coronary plaque in patients with or without susceptibility to unresolved thrombosis has different clinical implications [35]. From this perspective, coronary plaque healing acts as a safety net between plaque destabilization and ACS occurrence.

On the other hand, the presence of healed plaques has been associated with a higher prevalence of hyperlipidemia, diabetes mellitus, prior MI, more vulnerable plaque characteristics and amplified inflammation [23], indicating increased probability of recurrent plaque disruption. The high prevalence of newly formed layered patterns in ACS patients, as evidenced by Yi et al., aligns with this theory [40]. Moreover, repetitive episodes of subclinical thrombosis and subsequent healing are a mechanism of phasic plaque progression, indicative of accelerated obstructive coronary artery disease. It has even been argued that the repeated plaque destabilization and healing are one of the major drivers of atherosclerosis progression [40]. Indeed, the prevalence of healed plaques increases with increasing lumen area stenosis and is associated with more complex lesions [23, 37]. Evidence also suggests that culprit layered plaques in ACS patients are associated with low global coronary flow reserve (G-CFR), indicating coronary microvascular dysfunction [103]. According to a proposed pathophysiological mechanism, distal embolization of atherosclerotic debris and thrombi may contribute to the development of microvascular dysfunction [104]. It can therefore be speculated that atherosclerotic plaque disruption preceding the healing process releases microemboli that occlude the distal microvasculature.

Based on the above, coronary plaque healing preserves lumen patency and prevents ACS at the expense of lumen narrowing and an elevated risk of microcirculatory dysfunction. Thus, the presence of multilayered plaques should be carefully incorporated into future CAD prognostic tools, allowing for risk stratification reassessment and perhaps consideration of more intensive anti-inflammatory, antithrombotic and lipid-lowering therapy to prevent future MACE and to minimize the likelihood of rapid plaque progression. At present, the difficulty in detecting healed plaques remains one of the most significant limitations, as invasive endovascular imaging tools are required. For the same reason, current knowledge is largely derived from single time point studies. Technological advancements in endovascular imaging methods will facilitate the implementation of longitudinal studies, increase the sensitivity and specificity of layered plaque identification and finally support the integration of the concept of plaque healing capacity into prognostic models and clinical practice.

## Conclusion

A significant amount of coronary atherosclerotic plaque destabilizations do not cause clinical events. Plaque healing occurs and preserves lumen patency, preventing from ACS occurrence. Advancements in imaging modalities have facilitated the recognition of healed plaques in vivo as lesions with a layered pattern. The presence of this pattern has been associated with more vulnerable plaque features and possible worse prognosis, which might seem surprising at a glance, but with more careful consideration it is logically explained by the fact that healed plaques indicate an already burdened cardiovascular history. Therefore, plaque healing is both a safety mechanism and a hazard indicator. Researchers should focus more on developing new possible therapies that target impaired plaque healing, while clinicians ought to recognize the burdened history of patients with healed plaques and adjust their risk-stratification.

## Data Availability

No datasets were generated or analysed during the current study.
